# Transcriptional profiles of pulmonary artery endothelial cells in pulmonary hypertension

**DOI:** 10.1038/s41598-023-48077-6

**Published:** 2023-12-18

**Authors:** Navneet Singh, Carsten Eickhoff, Augusto Garcia-Agundez, Paul Bertone, Sunita S. Paudel, Dhananjay T. Tambe, Leslie A. Litzky, Katherine Cox-Flaherty, James R. Klinger, Sean F. Monaghan, Christopher J. Mullin, Mandy Pereira, Thomas Walsh, Mary Whittenhall, Troy Stevens, Elizabeth O. Harrington, Corey E. Ventetuolo

**Affiliations:** 1 Department of Medicine, Alpert Medical School of Brown University, Providence, RI USA; 2 Department of Computer Science, Brown University, Providence, RI USA; 3 Brown Center for Biomedical Informatics, Brown University, Providence, RI USA; 4 Department of Physiology and Cell Biology, College of Medicine, University of South Alabama, Mobile, AL USA; 5 Center for Lung Biology, College of Medicine, University of South Alabama, Mobile, AL USA; 6 Department of Pharmacology, College of Medicine, University of South Alabama, Mobile, AL USA; 7 Department of Mechanical Aerospace and Biomedical Engineering, College of Engineering, University of South Alabama, Mobile, AL USA; 8 Department of Pathology and Laboratory Medicine, University of Pennsylvania Perelman School of Medicine, Philadelphia, PA USA; 9 Department of Surgery, Alpert Medical School of Brown University, Providence, RI USA; 10 Lifespan Hospital System, Providence, RI USA; 11 Vascular Research Laboratory, Providence Veterans Affairs Medical Center, Providence, RI USA; 12 Department of Health Services, Policy and Practice, Brown University, Providence, RI USA

**Keywords:** Cell death, Translational research, Vascular diseases, RNA sequencing

## Abstract

Pulmonary arterial hypertension (PAH) is characterized by endothelial cell (EC) dysfunction. There are no data from living patients to inform whether differential gene expression of pulmonary artery ECs (PAECs) can discern disease subtypes, progression and pathogenesis. We aimed to further validate our previously described method to propagate ECs from right heart catheter (RHC) balloon tips and to perform additional PAEC phenotyping. We performed bulk RNA sequencing of PAECs from RHC balloons. Using unsupervised dimensionality reduction and clustering we compared transcriptional signatures from PAH to controls and other forms of pulmonary hypertension. Select PAEC samples underwent single cell and population growth characterization and anoikis quantification. Fifty-four specimens were analyzed from 49 subjects. The transcriptome appeared stable over limited passages. Six genes involved in sex steroid signaling, metabolism, and oncogenesis were significantly upregulated in PAH subjects as compared to controls. Genes regulating BMP and Wnt signaling, oxidative stress and cellular metabolism were differentially expressed in PAH subjects. Changes in gene expression tracked with clinical events in PAH subjects with serial samples over time. Functional assays demonstrated enhanced replication competency and anoikis resistance. Our findings recapitulate fundamental biological processes of PAH and provide new evidence of a cancer-like phenotype in ECs from the central vasculature of PAH patients. This “cell biopsy” method may provide insight into patient and lung EC heterogeneity to advance precision medicine approaches in PAH.

## Introduction

Endothelial dysfunction is a distinctive feature of pulmonary arterial hypertension (PAH)^1^. Controversy persists around the contribution of the endothelium in the proximal circulation versus the microvasculature, respective pulmonary arterial endothelial cell (PAEC) endotypes, and endothelial mechanisms responsible for disease progression. Inability to access human tissue in vivo has limited the validation of pre-clinical observations in pulmonary vascular disease. Most translational studies use human PAH endothelial cells (ECs) from lungs post-mortem or at the time of transplant or differentiated from inducible pluripotent stem cells^2^. While genomic studies of PAH cell lines from these sources have uncovered important mechanisms^3^, these methods are limited by selection bias (end-stage disease), the processing of cells after death or explant, or are derived from outside of the diseased organ. There are no data from living patients with PAH to inform whether differential gene expression of PAECs can discern disease subtypes or progression, identify therapeutic targets, or provide new insight into pathobiology.

We and others have previously reported successful isolation of cells with EC phenotype from the balloons of routine right heart catheters (RHC)^4–6^. We identified that patients with more severe PAH were more likely to have successful propagation of cells and that PAH PAECs may have been resistant to anoikis, dysregulated programmed cell death when a cell is detached from the extracellular matrix (ECM). Limitations of this work included the possibility of phenotypic drift with serial passaging and the lack of additional molecular and functional studies.

The aims of this study were to use RNA sequencing to provide further validation of this method and to perform additional PAEC phenotyping including over the PAH clinical disease course. First, we evaluated changes in the transcriptome of serial in vitro passages of PAECs. Second, we compared the PAH gene expression signature to that of controls, among repeated samples in the same patients over time and compared PAH gene expression to that of pre-capillary pulmonary hypertension (PH) in non-Group 1 disease. Based on our prior observations, we explored anoikis resistance as a novel paradigm that may contribute to the propagation of pulmonary vascular disease.

## Results

### Cohort characteristics

Forty-nine subjects had a total of 54 RHCs over the course of the study (two subjects had a total of seven RHCs with samples obtained at each catheterization). To evaluate the effect of serial passaging on the transcriptome, six passaged specimens (passage 3 and 4 for each sample) from three of the 54 unique catheter tips were sequenced. Characteristics of subjects are presented in Table 1 and Table S1. The median age was 63 years (range 19–91 years) and 37 (69%) were female. Twenty (37%) balloon tips were from subjects with PAH, the majority of whom had idiopathic PAH (IPAH), connective tissue disease-associated PAH (CTD-APAH) and portopulmonary hypertension (PoPH). Most PAH patients were prevalent and were receiving PAH specific medications at the time of RHC. Six (30%) were treatment naive. The average pulmonary vascular resistance (PVR) was 4.0 Wood units (range 1.2–19.6 Wood units). Eighteen (33%) were from Group 2 PH patients, of which five (9%) had combined pre- and post-capillary PH. Eight tips (15%) were from subjects with Group 3 PH, half of whom had obstructive lung disease. One tip was from a subject with chronic thromboembolic (Group 4) PH, four from subjects with Group 5 PH and three from patients who underwent RHC but did not have evidence of PH, designated as controls.

**Table 1:** Characteristics of subjects, by balloon tips.

Balloon tips*, n	54
Subjects, n	49
Female sex	37 (69)
Age, years	63 (19–91)
Body mass index, kg/m^2^	28 (16–46)
Self-reported race	
White	42 (77)
Black	9 (17)
Asian	1 (2)
Other	5 (9)
Self-reported Hispanic ethnicity	7 (13)
Pulmonary hypertension subtypes	
Group 1 PAH	20 (37)
Idiopathic PAH	5 (9)
Heritable PAH	2 (4)
CTD-APAH	5 (9)
Congenital heart disease-APAH	2 (4)
HIV-APAH	1 (2)
Portopulmonary hypertension	5 (9)
Group 2 PH	18 (33)
Combined pre-post capillary PH	5 (9)
Group 3 PH	8 (15)
Group 4 PH	1 (2)
Group 5 PH	4 (7)
Control^†^	3 (6)

Data represented as n (%) or median (range). *Two subjects had a total of seven biological replicates (one subject with two balloon tips, one subject with five balloon tips). ^†^Control are subjects who underwent right heart catheterization without evidence of PH. *PAH* pulmonary arterial hypertension, defined as mean pulmonary artery pressure > 20 mmHg, pulmonary capillary wedge pressure < 15 mmHg, and pulmonary vascular resistance > 3.0 Woods units, *PH* pulmonary hypertension, defined as mPAP > 20 mmHg, *CTD* connective tissue disease, *HIV* human immunodeficiency virus, *APAH* associated pulmonary arterial hypertension.

### The transcriptome remained stable over serial passaging of PAECs

All RNA sequencing was performed on passage three or four for all samples. Unsupervised sequencing data of passaged specimens were analyzed to investigate whether the transcriptome changes meaningfully with early passaging. We compared passage 3 with passage 4 in PAECs from three subjects with heritable PAH (HPAH), PoPH and Group 2 PH. Cosine similarities were computed between a subject’s sequencer output and the corresponding passaged sequence. Pairs were highly similar (range 0.95–0.99) and the average similarity to all other samples (from other PAEC lines) was low (range 0.79–0.86) (Table S2 and Fig. S1). Passage number was fixed for all comparisons (PAH vs. control, across biological replicates).

### PAECs from PAH subjects differentially expressed genes that regulate the oxidative stress response, alter cellular metabolism and participate in BMP and Wnt signaling pathways

**Figure 1:**
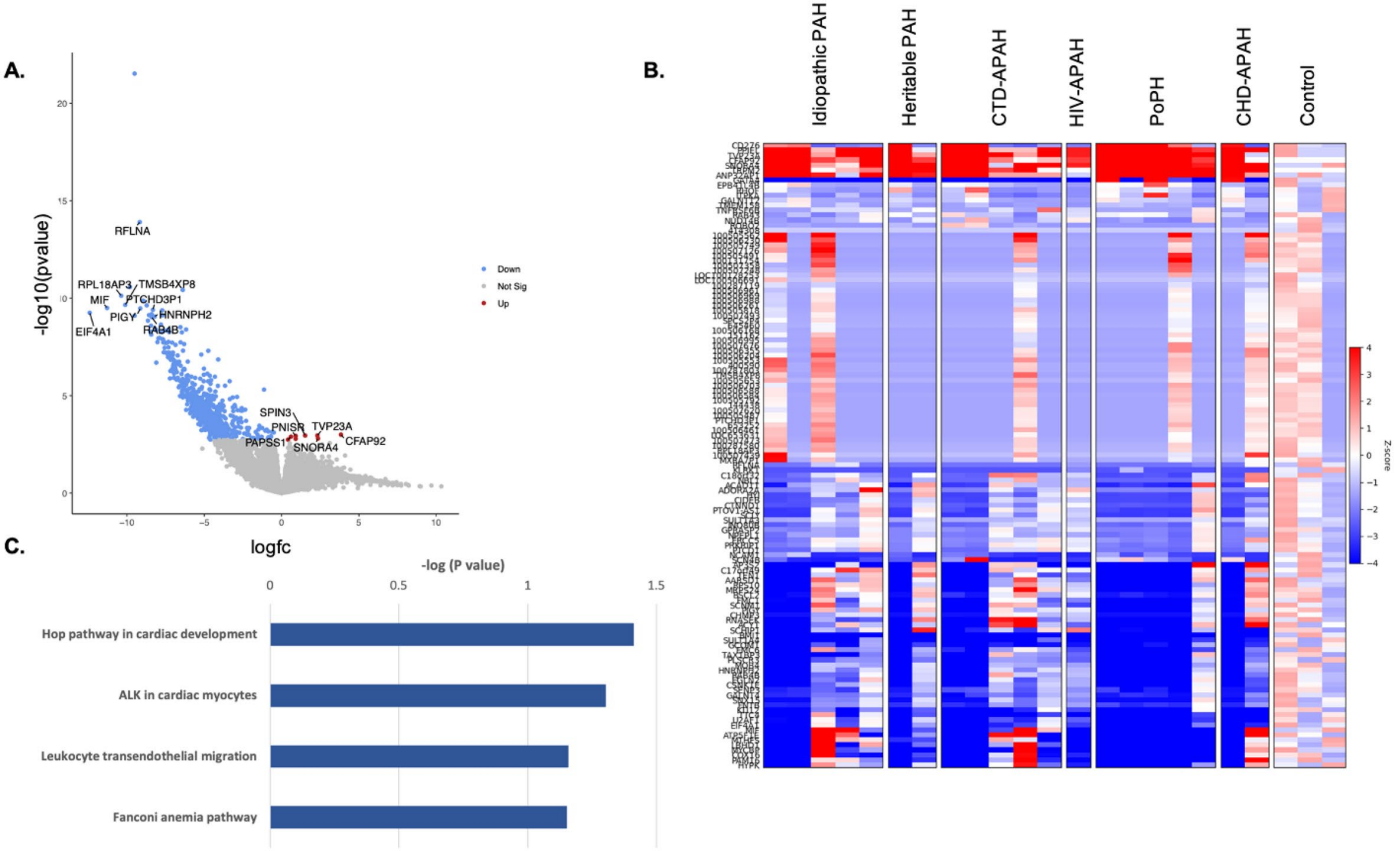
Differential gene expression in Group 1 pulmonary arterial hypertension vs. controls without pulmonary hypertension. (**A**) Volcano plot of fold-change in differential gene expression: Group 1 pulmonary arterial hypertension vs. controls.* (**B**) Heatmap of top differentially expressed genes. (**C**) Biologic pathways mapped from differentially downregulated genes. *p-values adjusted for multiple comparisons based on a false discovery rate (FDR) < 0.05. A p_FDR_ < 0.05 was considered significant. *PAH* pulmonary arterial hypertension. *CTD* connective tissue disease. *HIV* human immunodeficiency virus. *PoPH* portopulmonary hypertension. *CHD* congenital heart disease. *APAH* associated pulmonary arterial hypertension.

An unsupervised analysis was performed of the full transcriptome of PAECs from subjects with PAH as compared to controls. Fold-change analysis demonstrated 667 differentially expressed genes in PAH subjects as compared to controls (Fig. 1A,B). Six genes were significantly upregulated (p_FDR_ < 0.05) in PAH subjects: *CFAP92*, *SNORA4*, *TVP23A*, *SPIN3, PNSIR* and *PAPSS1* (Table 2).

**Table 2:** Differentially regulated genes and ontology: Group 1 pulmonary arterial hypertension vs. controls.

Top differentially upregulated genes and ontology				
Symbol	Gene name	logFC	Adj. p-value	Ontology
*CFAP92*	Cilia and flagella associated protein 92 (putative)	3.84	0.03	Aberrant expression associated with deficiency of Acyl-CoA Dehydrogenase (the first enzyme involved in mitochondrial fatty-acid oxidation), associated with mitochondrial Complex I deficiency
*SNORA4*	Small nucleolar RNA, H/ACA box 4	2.36	0.04	Involved in RNA processing, 17β-estradiol results in increased *SNORA4* mRNA, snoRNAs are involved in cancer pathogenesis
*TVP23A*	Trans-golgi network vesicle protein 23 homolog A	2.32	0.03	Protein secretion, vesicle-mediated transport, vacuole, integral component of Golgi membrane, plasma membrane, enhanced protein expression in many types of cancers
*SPIN3*	Spindlin family member 3	1.53	0.03	Methylated histone binding, regulation of transcription
*PNISR*	PNN interacting serine and arginine rich protein	0.91	0.03	Participates in RNA binding, active in presynaptic active zone
*PAPSS1*	3′-Phosphoadenosine 5′-phosphosulfate synthase 1	0.61	0.04	Bifunctional enzyme: transfer of sulfate to ATP and transfer of phosphate from ATP to APS, required for biosynthesis of sulfated L-selectin ligands in endothelial cells

Differentially downregulated genes and biologic pathways				
Gene	Ontology			
Hop pathway in cardiac development				
*GATA4*	Transcription factor in BMP pathway, transcriptional activator of ANF in cooperation with *NKX2-5*			
*NKX2-5*	Transcriptional activator of ANF in cooperation with *GATA4*			
ALK in cardiac myocytes				
*GATA4*	Transcription factor in BMP pathway, transcriptional activator of ANF in cooperation with *NKX2*-5			
*NKX2-5*	Transcriptional activator of ANF in cooperation with *GATA4*			
*ACVR1*	BMPR1, transduces signals of BMPs, forms a complex with BMPs (including BMPR2) and then recruits SMAD, activates canonical SMAD signaling. phosphorylates SMAD1/5/8, suppresses TGFbeta/activin pathway,			
Leukocyte transendothelial migration				
*ACTN1*	Actin filament bundle assembly, platelet degranulation, regulation of Bcl-2-apoptotic process			
*CTNND1*	Delta-catenin, negative regulation of Wnt signaling pathway (differentiation, proliferation, migration), cell–cell adhesion,			
*RAC2*	Augments production of ROS by NOX1 and 2, positive regulation of proliferation			
*VASP*	Substrate of BCR-ABL oncoprotein, actin-associated protein, cell migration, platelet activation, cytoskeleton remodeling, actin filament elongation			
Fanconi anemia pathway				
*SLX1A*	DNA endonuclease			
*SLX1B*	DNA endonuclease			
*CENPS*	DNA binding component of the Fanconi Anemia core complex, leads to monoubiquitination of the complex in response to DNA damage, prevents chromosomal breakage			

Six-hundred and sixty-one RNA transcripts were differentially downregulated in PAH subjects compared to controls; 234 mapped to known genes. Pathway analysis revealed four pathway clusters: Hop pathway in cardiac development, ALK in cardiac myocytes, leukocyte transendothelial migration and Fanconi Anemia pathway (Fig. 1C). Downregulated genes and ontologic themes related to bone morphogenetic protein (BMP) signaling (*GATA4*^7^, *NKX2-5*^8^, *ACVR1*^9^), canonical Wnt signaling (*CTNND1*), production of ROS by NADPH oxidase (*RAC2*), regulation of apoptosis (*ACTN1*) and posttranslational ubiquitination (*CENPS*^10^) are described in Table 2.

### PAECs from subjects with PAH exhibited resistance to anoikis

**Figure 2:**
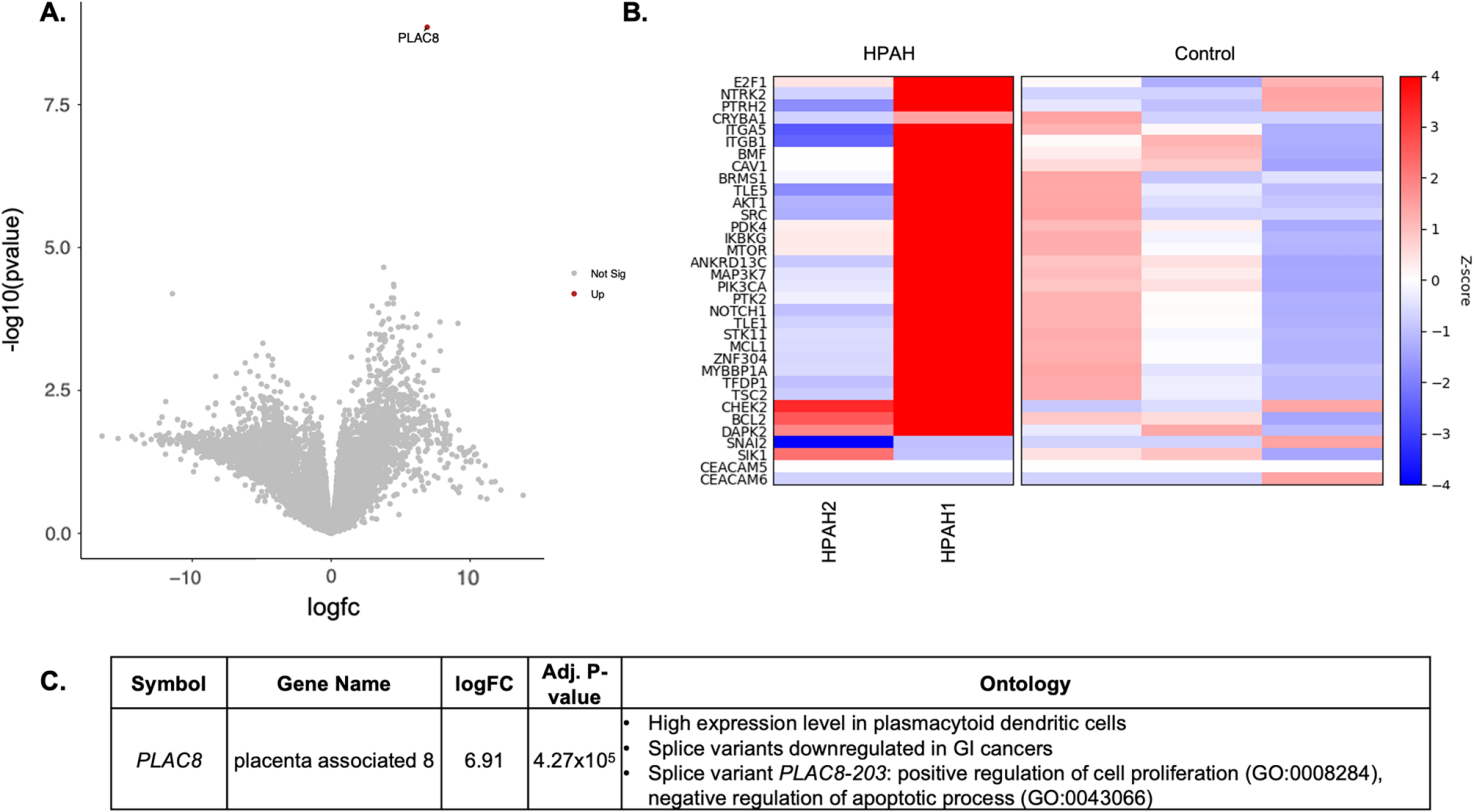
Differential gene expression in subjects with heritable pulmonary arterial hypertension (HPAH) versus controls. (**A**) Volcano-plot of unsupervised fold-change analysis of differentially expressed genes between HPAH1 vs controls*. (**B**) Heatmap of genes related to anoikis (GO: 0043276) demonstrated significant between-subject heterogeneity with increased expression in HPAH1, who has classical heritable PAH, and equivocal expression in HPAH2, who has hereditary hemorrhagic telangiectasia. (**C**) In an unsupervised analysis, one gene was differently expressed greater in PAECs from HPAH1 as compared to controls (*PLAC8*) with a splice-variant (*PLAC8-203*) identified as responsible for positive regulation of cell proliferation and negative regulation of apoptosis. *p-values adjusted for multiple comparisons based on a false discovery rate (FDR) < 0.05. A p_FDR_ < 0.05 was considered significant.

In our initial publication^4^, we were most likely to propagate primary cells from patients with severe PAH. We noted that PAECs derived from certain PAH patients were capable of rapid expansion and growth with traditional passaging in culture media and continued to expand outside of a monolayer even when transferred to a plastic substrate, suggesting ex vivo replication competency. PAECs from two subjects with HPAH with qualitative evidence of replication competency had RNA sequencing performed. Subject HPAH1 has classical HPAH but declined genetic testing and subject HPAH2 is known to have an *ALK1* mutation and hereditary hemorrhagic telangiectasia^11^. A heatmap of pre-selected genes that govern anoikis (GO: 0043276) of the HPAH specimens compared to controls is shown in Fig. 2B and demonstrated between-subject heterogeneity of expression in genes related to anoikis as compared to controls. Most genes related to anoikis were upregulated in HPAH1 whereas HPAH2 had more equivocal gene expression. Fold-change analysis of differentially expressed genes in HPAH1 vs. controls revealed increased expression of *PLAC8* (Fig. 2A,C)^12^ whereas despite known *AKL1* mutation, bulk gene expression of HPAH2 was similar to controls (Fig. S2). Fold change analysis of differential gene expression between the two HPAH specimens demonstrated significantly different genomic expression (Fig. S3).

**Figure 3:**
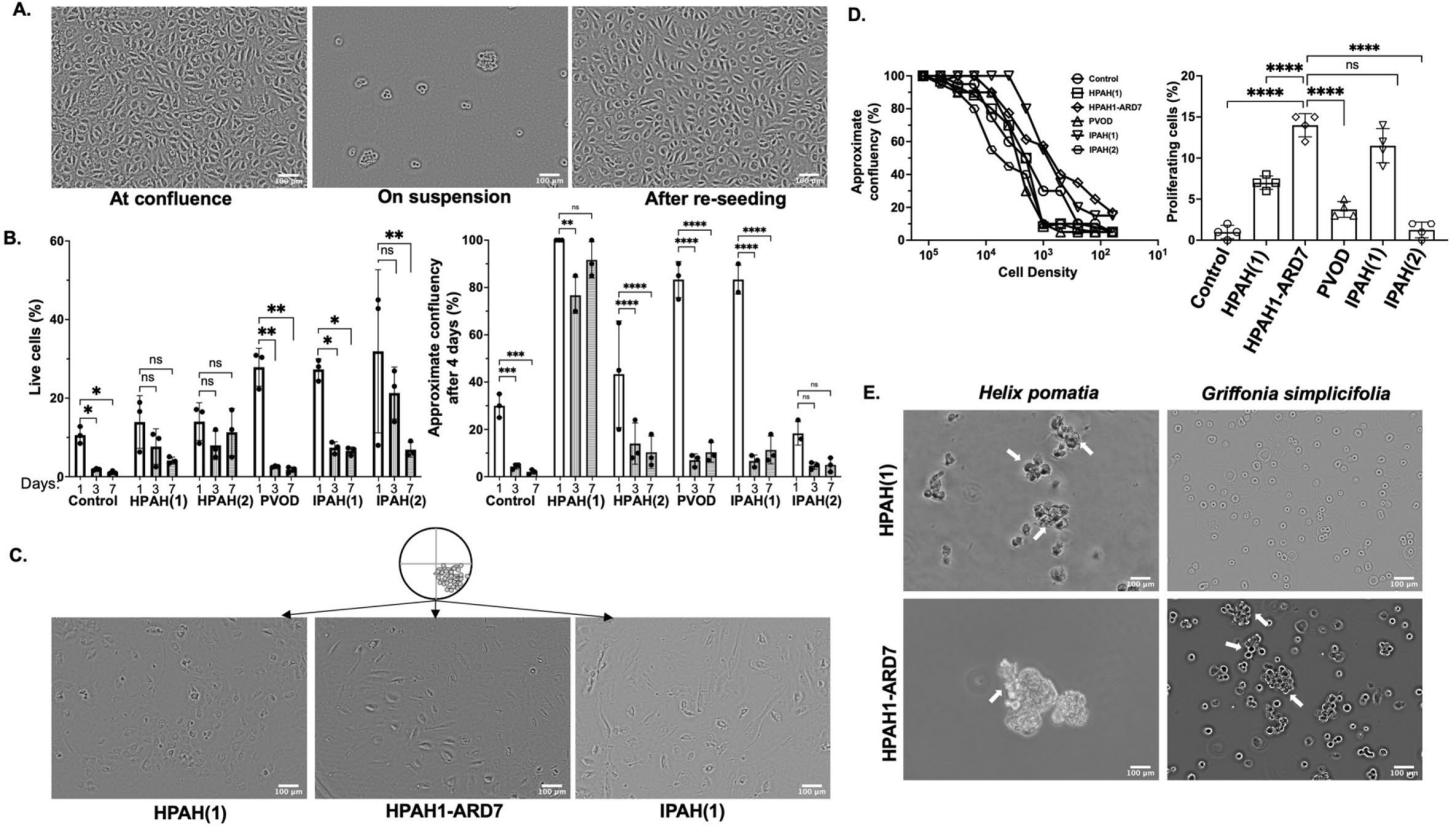
PAECs isolated from patients with pulmonary hypertension exhibit anoikis resistance as compared to commercial controls. (**A**) Some PAECs survive in suspension over 7 days and proliferate when reseeded on gelatin-coated plastic culture dishes, characteristic of anoikis resistance. Phase-contrast images of PAECs from a patient with hereditary pulmonary arterial hypertension (HPAH1) are shown, including cells at confluence (left-hand panel), 7 days following suspension in ultra-low attachment 6-well plates (middle panel), and 8 days after re-seeding the cells in suspension on a culture dish (right-hand panel). (**B**) The growth of anoikis-resistant cells is distinctive among patients with pulmonary hypertension. ~ 10–30% of HPAECs survived in suspension for 24 h, whereas ~ 5% of cells survived for 3- and 7-days (left-hand panel). The right-hand panel demonstrates percent confluency on day 4 (y-axis) of cells that have been in suspension for 1, 3 and 7 days (x-axis). Anoikis-resistant cells from one HPAH patient (HPAH1) exhibited rapid proliferation, whereas the growth capacity of anoikis-resistant cells from patients pulmonary veno-occlussive disease (PVOD), idiopathic PAH (IPAH1), IPAH2, and a second HPAH patient (HPAH2) was limited. (**C**) Representative phase-contrast images taken on the 14th day of incubation of the single-cell cloning wells from two different subjects are shown. The insert on the top demonstrates that the colony size in each well occupied less than 25% of the well. HPAH1 cells (left-hand panel), anoikis-resistant PAECs collected after 7 days in suspension from HPAH1 (HPAH1-ARD7, middle panel), and IPAH1 cells are shown. IPAH1 cells did not survive after 7 days in suspension. (**D**) Low density and single-cell proliferation are variable among PAECs isolated from patients with pulmonary hypertension. Confluency of serially diluted cells from six different cell types (Control, HPAH1, HPAH1-ARD7, PVOD, IPAH1, and IPAH2) is plotted after 10 days of culture, with densities ranging from 125K/well to 62 cells (left-hand panel). Anoikis-resistant cells are more replication-competent in single-cell cloning assays, 2 weeks after single-cell cloning (right-hand). (**E**) Lectin-induced agglutination discriminates endothelial cell phenotypes in vitro. *Helix pomatia* agglutinates PAECs, but not pulmonary microvascular endothelial cells. *Helix pomatia* agglutinated HPAECs from HPAH1, whereas *Griffonia simplicifolia* did not. *Griffonia simplicifolia* agglutinates pulmonary microvascular endothelial cells and endothelial progenitor cells. Here, *Griffonia simplicifolia* agglutinated anoikis resistant, highly proliferative cells from HPAH1-ARD7. Arrows indicate agglutinated cells. Data in panels (**B**) and (**C**) represent technical replicates for PH patients and n = 3 for commercial control. All phase-contrast images are taken with a 10 × objective, and the scale bar represents 100 μm. *HPAH* heritable pulmonary arterial hypertension. *PVOD* pulmonary vent-occlusive disease. *IPAH* idiopathic pulmonary arterial hypertension.

To further explore across-subject heterogeneity in the anoikis resistance paradigm, we selected additional PAEC samples from PAH patients of varied clinical phenotypes. Serial dilution was performed on the two HPAH samples above (HPAH1 and HPAH2), three additional PAH PAEC samples, and three purchased commercial controls (Lonza; Basel, Switzerland). PAECs from all five subjects exhibited a proliferative capacity in low density and single-cell assays, to varying degrees; approximately 5% of the cell colonies survived the suspension, characteristic of anoikis-resistance (Fig. 3A,B, left-hand panel). The growth of anoikis-resistant clones varied and was more robust in certain clinical phenotypes (HPAH1 and IPAH1 vs. pulmonary veno-occlusive disease [PVOD]) and in untreated (incident) disease (IPAH1 vs. IPAH2) but was not necessarily related to hemodynamic burden at the time of RHC (Fig. 3D, Table S3). PAECs from HPAH1 survived in suspension without ECM attachment for 7 days, whereas the PAECs from IPAH1, IPAH2, PVOD and HPAH2 demonstrated more limited replication competency (Fig. 3B, right-hand panel, C). The anoikis-resistant cells from HPAH1 formed clusters in the presence of *Griffonia* lectin, characteristic of an endothelial microvascular or progenitor-like phenotype (Fig. 3E)^13^.

### Transcriptome changes in biological replicates tracked with disease course

**Figure 4:**
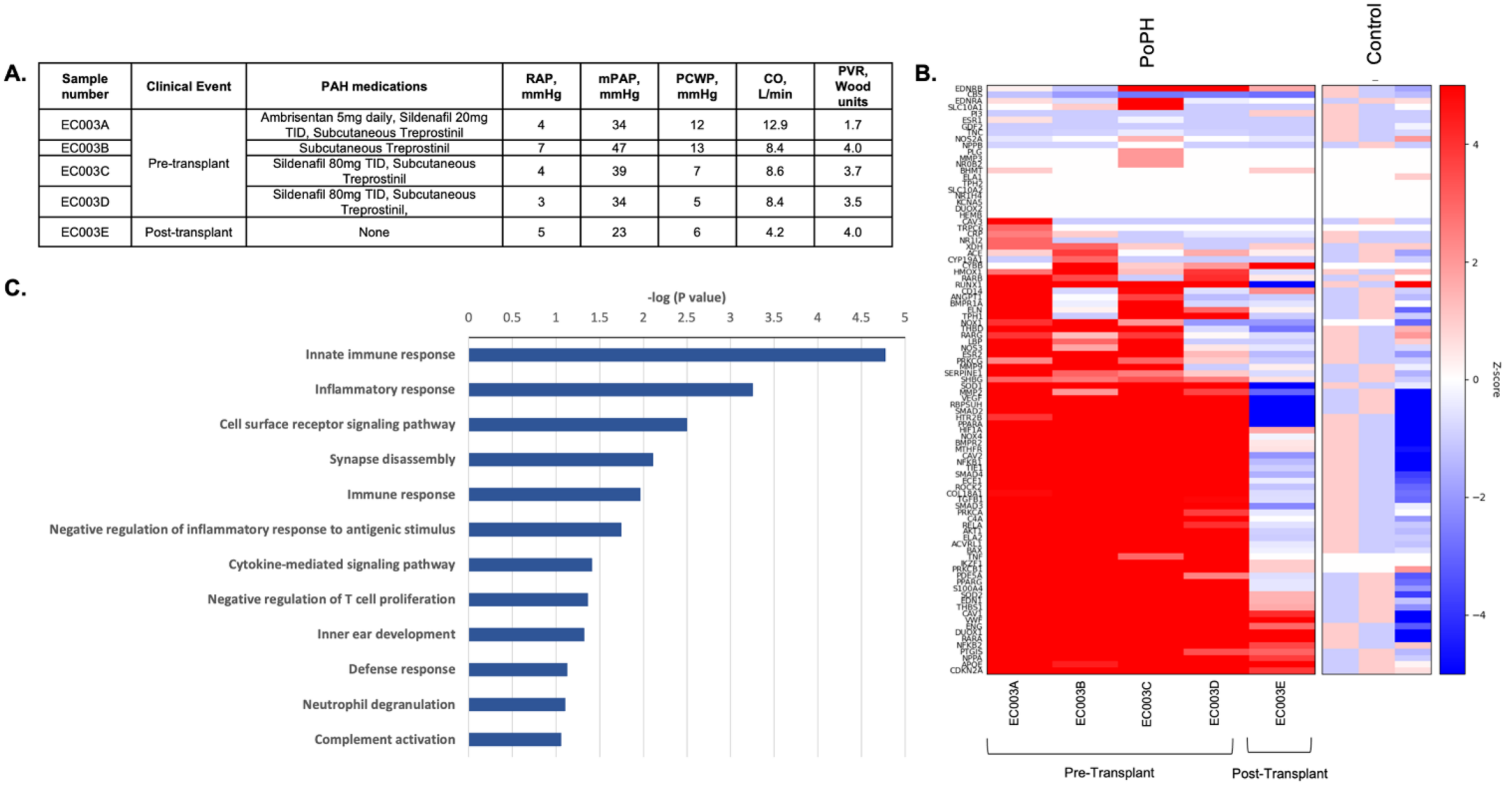
Biological replicates in a subject with portopulmonary hypertension. (**A**) Hemodynamics before and after liver transplant. Near resolution of pulmonary hypertension was seen after liver transplantation off all PAH therapy. (**B**) Heatmap of differential gene expression in genes related to portopulmonary hypertension (gene list assembled from Roberts, K., Kawut, S., et al. AJRCCM. 2009). A “normalization” of gene expression toward control levels was seen after liver transplantation (**C**) DAVID was used to mine GO biologic pathways of downregulated genes of pre-transplant endothelial cells as compared to post-transplant cells. Pathways related to the immune response were downregulated pre-transplant as compared to post-transplant. *RAP* right atrial pressure, *mPAP* mean pulmonary artery pressure, *PCWP* pulmonary capillary wedge pressure, *CO* cardiac output, *PVR* pulmonary vascular resistance, *TID* three times daily. *PoPH* portopulmonary hypertension.

Two subjects had biological replicates obtained from serial catheterizations and had major clinical events during this time. The first subject was a 56-year-old female diagnosed with PoPH due to primary biliary cirrhosis and started on PAH therapy. PVR improved but she remained in a high cardiac output state. Following liver transplantation, repeat RHC after she had been weaned off all PAH therapy showed that her PH had nearly resolved (Fig. 4A). A heatmap of pre-selected genes related to the pathogenesis of PoPH^14^ is shown in Fig. 4B with genes differentially expressed compared to controls over serial samples. Comparison of the transcriptome before and after transplant demonstrated a marked reversal of gene expression away from the PAH pattern and closer to that of control subjects after transplant. Transplant-induced changes in the PAEC transcriptome clustered to biological pathways related to the immune response (Fig. 4C)^15^.

**Figure 5:**
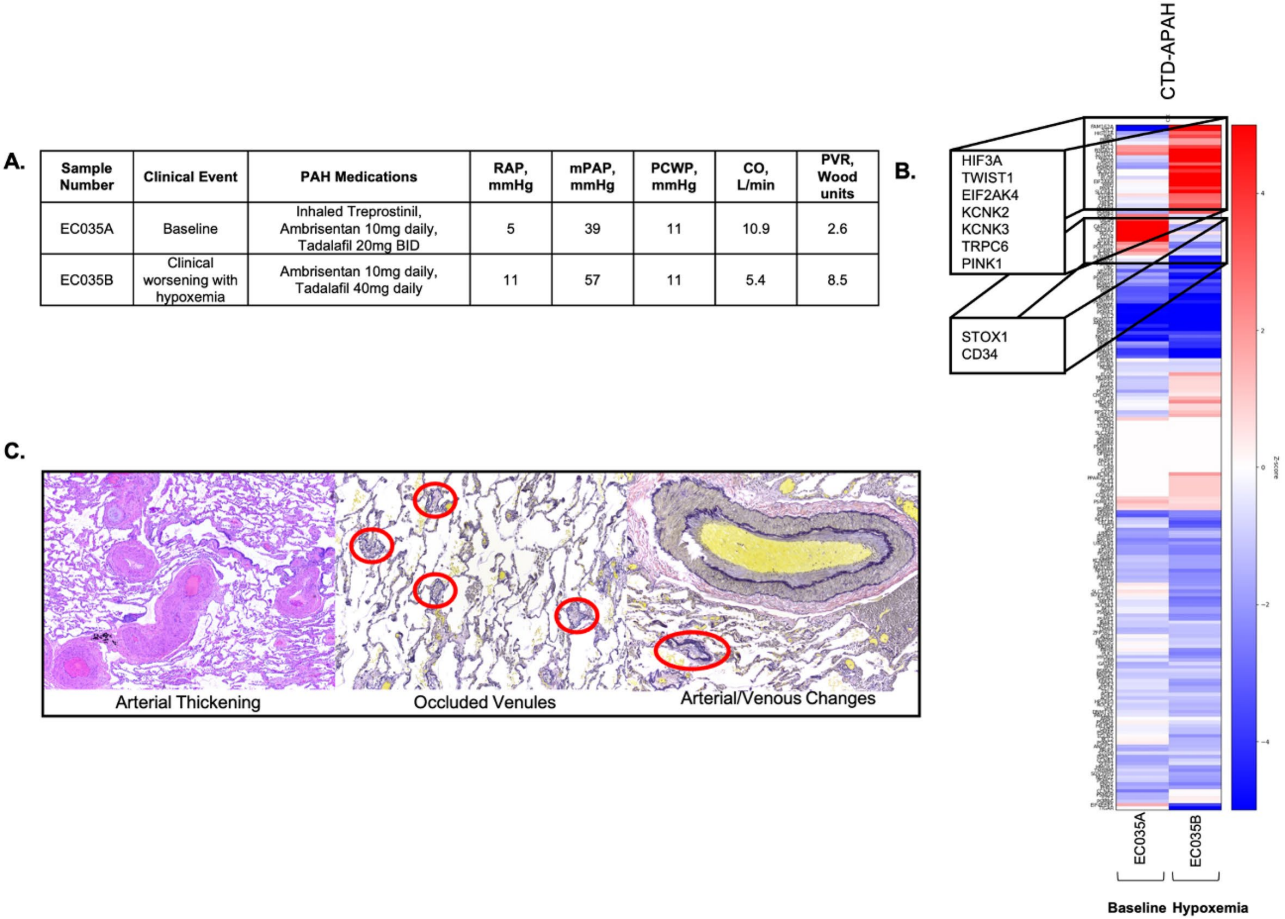
Biological replicates in a subject with systemic sclerosis-associated pulmonary arterial hypertension. (**A**) Hemodynamics at baseline and clinical worsening. The subject’s clinical course was marked by profound hypoxemia at the time of the second right heart catheterization; the development of pulmonary veno-occlusive disease (PVOD) was suspected clinically. (**B**) Heatmap of differential expression of genes related to PVOD and hypoxia showed increased expression of genes including *HIF-3α, TWIST1* and *EIK2AK4* along with decreased expression of *STOX1* and *CD34* (gene list assembled from Hypoxia (GO: 0071456) and *EIF2AK4*). (**C**) Histology of explanted lung demonstrating venule involvement.  *RAP* right atrial pressure. *mPAP* mean pulmonary artery pressure. *PCWP* pulmonary capillary wedge pressure. *CO* cardiac output. *PVR* pulmonary vascular resistance.

The second subject, a 44-year-old male with CTD-APAH due to systemic sclerosis, experienced clinical worsening characterized by increasing PVR and profound hypoxemia (Fig. 5A). Nine months after his second RHC the patient developed hypoxemic respiratory failure requiring lung transplantation. Histopathologic examination of the explanted lungs revealed significant pulmonary venule involvement (Fig. 5C). A heatmap of pre-selected genes related to hypoxia (GO: 0071456) and *EIF2AK4* showed marked changes between the two catheterizations and compared to controls (Fig. 5B). Comparison of the transcriptome between these two samples (baseline and clinical worsening) did not reveal statistically significant differences. However, expression was increased in *HIF-3α, TWIST1, EIF2AK4*, and *PINK1* and decreased in *STOX1* and *CD34*.

### Subjects with group 2–5 precapillary PH demonstrated similar transcriptional profiles as group 1 PAH

**Figure 6:**
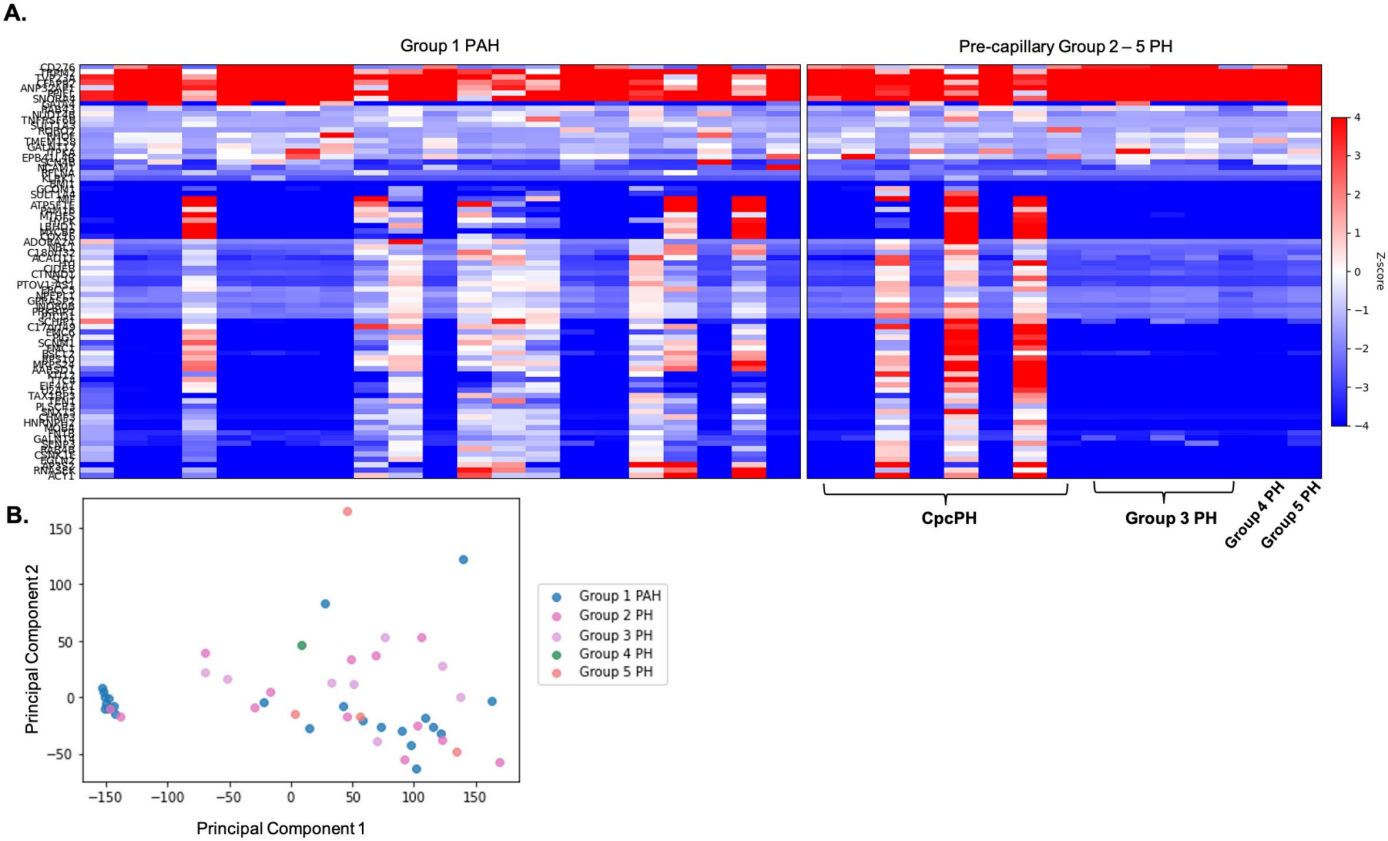
Gene expression is similar across all subjects with pre-capillary pulmonary hypertension. (**A**) Heatmap and (**B**) principal component analysis (PCA) plot of differential gene expression between subjects with Group 1 pulmonary arterial hypertension (PAH) vs Group 2–5 precapillary pulmonary hypertension (PH), defined as mPAP > 20 mmHg, a PCWP ≤ 15 mmHg, and a PVR ≥ 3 Wood units. Despite within group heterogeneity, there appears to be similarities in gene expression between Group 1 PAH and precapillary Group 2–5 PH and there was no evidence of differential gene expression across precapillary PH from Group 1 versus Group 2–5. *mPAP* mean pulmonary artery pressure, *PCWP* pulmonary capillary wedge pressure, *PVR* pulmonary vascular resistance, *CpcPH* combined pre-post capillary pulmonary hypertension.

The transcriptome of PAECs from Group 1 PAH subjects was compared with PAECs from all Group 2–5 precapillary PH (mPAP > 20 mmHg, PCWP ≤ 15 mmHg, and PVR ≥ 3 Wood units). A representative heatmap and principal component analysis (PCA) plot of the top differentially expressed genes is shown in Fig. 6, with similar patterns noted between the two groups. An unsupervised fold-change analysis of the full transcriptome failed to find significant differences in expressed genes between Group 1 PAH and all Group 2–5 precapillary PH subjects (i.e., transcriptional signatures among PAECs from all subjects with pre-capillary PH were similar).

## Discussion

We have shown that procurement of PAECs from routine RHC is a valid method that provides a window into the progression of pulmonary vascular disease from living patients over time. Transcriptomic changes appeared stable over early passages with a signature from the central pulmonary vasculature that is characteristic of PAH, but also provides new insights. PAECs from subjects with PAH demonstrated differential expression of genes involved in BMP and Wnt signaling, cancer pathogenesis, fatty acid oxidation and glycolysis. Select PAECs from PAH patients were replication competent and resistant to anoikis, a novel pathologic mechanism that warrants further evaluation.

The genetic signature of PAH PAECs recapitulated several established and fundamental pathways in PAH, supporting our hypothesis that the proximal circulation is involved in pulmonary vascular disease and requires dedicated study. We demonstrated alteration in the transcription of genes related to BMP^16^ and canonical Wnt signaling^17^ in PAH subjects. There was downregulation of *GATA4* and *NKX2-5*, which together work as transcriptional activators of atrial natriuretic factor (ANF)^18^, implicated in experimental PH^19^. *GATA4* functions as a transcription factor in the BMP signaling pathway^18^ and plays a role in serotonin-induced proliferation in PAH^20^. *ACVR1* (also *BMPR1* or *ALK2*) was downregulated in PAH PAECs, which aligns with established dysfunctional BMP/TGF-β signaling in experimental PH^21^ and is a new therapeutic target in PAH^22^. *CTNND1*, which encodes for delta-catenin and is an important negative regulator within the canonical Wnt signaling pathway, was downregulated in PAECs from PAH subjects; downregulation of beta-catenin and canonical Wnt signaling has previously been linked to endothelial proliferation in PAH^23^, observed in a monocrotaline model of PH^24^, and tied to carcinogenesis^25^. Together, these observations support the study of cells from the more proximal vasculature as an accessible, valid and informative source of tissue from living PAH patients.

Anoikis deregulation is a hallmark of metastasis and occurs in ECs^26^ but has not been described in pulmonary vascular disease. Our functional assays demonstrated populations of PAH PAECs that maintained replication competency despite low density media, dilutional cloning and without ECM attachment. Their enhanced ability to replicate may be due to increased expression of *PLAC8.* This gene has a known splice variant that has been shown to promote cell proliferation and negatively regulate apoptosis^27^. We speculate that upregulation in most genes related to anoikis represents a compensatory response to the anoikis resistance we observed in functional assays or that we may be isolating a discrete subpopulation of cells involved in the injury/repair response. Apoptosis resistance has been demonstrated in rat microvascular ECs^28^ and in healthy human microvascular ECs exposed to shear stress^29^, but the role of apoptosis in EC endotypes in human PAH remains controversial^30^. Findings from the present study lend support to the hypothesis that enhanced PAEC proliferation with a cancer-like phenotype contributes to vascular remodeling in PAH and that this hyperproliferative phenotype exists in proximal ECs. Importantly, we noticed significant between-subject heterogeneity in both bulk sequencing, focused pathway analysis and in functional assays. This highlights the observation that current clinical classifications do not accurately capture the differences among patients that precision methods do. While additional studies are needed to understand endothelial and progenitor-like cross-talk as well as microheterogeneity within and across the pulmonary circulation, anoikis resistance may serve as a new paradigm to understand PAH.

Several themes related to long-hypothesized but less established mechanisms of PAH emerged in our study including oxidative stress^31^, alterations in the mitochondrial electron transport chain^32^ and fatty acid oxidation^33^, sex-hormone signaling^34^, and maladapted post-translational modification^35^. These purported pathways have been difficult to translate from preclinical models to human PAH. Oxidative stress is theorized to alter cellular senescence, necrosis and apoptosis in PAH^36^, but the exact mechanisms remain controversial^37^. Reactive oxygen species (ROS) stimulate PAEC proliferation^38^, trigger angiogenesis^39^, and activate HIF-1α, a master regulator in PH^40^. *RAC2* encodes for a Rho-GTPase important for canonical Wnt signaling^25^, but is also required for ROS production by NADPH Oxidases-1 and -2. Mitochondrial dysfunction plays a central role in PAH with a shift toward aerobic glycolysis^41^ although exact mechanisms remain elusive. In our PAH subjects, *CFAP92* was upregulated, a gene noted to be associated with deficiencies of both Acyl-CoA Dehydrogenase, the first enzyme in mitochondrial fatty acid oxidation, and Complex I of the electron transport chain^42^. We confirm a role for metabolic dysfunction and oxidative stress in human patients living with PAH.

PAECs from PAH subjects demonstrated upregulation of *SNORA4*, an mRNA known to be upregulated by 17-β-estradiol and belonging to a family of RNAs implicated in cell proliferation, angiogenesis, and metastasis in cancer models^43, 44^. PAH is a sexually dimorphic disease and sex steroids have been a major focus of experimental and observational studies^45^; we and others are currently conducting clinical trials to target estradiol and supplement dehydroepiandrosterone (NCT03648385, NCT03229499, NCT03528902). *CENPS*, which codes for an anti-centromere protein, was downregulated in PAEC lines from PAH subjects. Anti-centromere proteins are well recognized to play a role in tissue fibrosis in CTDs including systemic sclerosis^46^. *CENPS* appears to also play a role in ubiquitination, a posttranslational modification process that is maladapted during PAH pathogenesis^47^. Ubiquitin proteosome function has emerged as a novel therapeutic target in experimental PH^48^, but evidence in human disease has not previously been described.

PoPH is a poorly understood and morbid sub-type of PAH that, for unclear reasons, can be cured with liver transplantation. Serial samples from a subject with PoPH demonstrated transcriptional changes in biologic pathways related to the immune response and inflammation that “normalized” after liver transplant, albeit in the setting of typical immunosuppressive medications after transplant. Pathways responsible for negative regulation of the inflammatory response and T-cell proliferation were downregulated, suggesting unchecked inflammation in PoPH and/or cirrhosis. These observations for the first time provide a molecular window during the clinical course of PoPH that includes the impact of liver transplant (and/or related immunomodulatory medications) on the pulmonary vasculature. Replicates from a subject with CTD-APAH found to have histopathological evidence of PVOD demonstrated transcriptional changes in genes that govern the response to hypoxia (e.g., *HIF-3α*), a hallmark clinical sequela in patients with PVOD. Interestingly, *EIF2AK4* was *upregulated* as the subject worsened, despite known loss of function in PVOD^49^. Serial measurements of EC *EIF2AK4* gene expression during PVOD have never been described; as with anoikis, it is possible that increased expression may represent a compensatory response or that we have captured a sub-population of reparative cells. These discrepant observations underscore that additional mechanistic work is needed alongside studies of pulmonary vascular EC heterogeneity.

Studies have previously demonstrated more phenotypic similarities across PH Groups than within them^50–52^, leading to efforts to subphenotype pulmonary vascular disease^53^. In our study, subjects with precapillary PH had similar transcriptional profiles and we failed to find evidence of differential gene expression across these groups (Group 1 PAH vs. precapillary Group 2–5 PH). Two subjects with known HPAH had distinct gene expression from each other and only one was unique from controls. This supports the argument that similar molecular processes may underpin EC dysfunction and vascular remodeling in patients with precapillary disease, regardless of clinical designation, and there is significant within-group heterogeneity in PAH, timely observations given efforts to refine (and possibly dismantle) clinical classification using precision-based approaches.

This study has limitations. It is not possible to prove that cultured cells are from the pulmonary artery, however the balloon is wedged only in the pulmonary arteries where it has the greatest and longest contact with the pulmonary artery wall. We have previously published evidence of typical EC surface expression markers and several balloons advanced to the right ventricle did not yield results^4^. The genetic signature of ECs may change with passaging. We observed transcriptome stability in early passages and all analyses were across fixed and early passage numbers. Work is underway to characterize within-sample heterogeneity from fresh samples using single-cell sequencing and to compare the signature of harvested PAECs to that of circulating progenitors. Performing this analysis on the primary cell population from the balloon tips will help to address the concern that we are selecting for the most proliferative ECs with early passaging. The imbalance in our clinical groups including controls (who underwent RHC for clinical evaluation and thus are not pure “healthy controls”) may have created bias, however our findings are highly relevant to PAH pathobiology, providing face validity. The downregulation of many genes in PAH cell lines is inconsistent with observations by other groups^54–56^, however we submit that proximal PAECs from living patients with PAH is a unique population of cells that may not be directly comparable to prior studies. We do not know the mutation status of most of the patients who provided samples (including HPAH1, as they have declined genetic testing). The clinical event rate was relatively low during follow-up (three deaths among subjects with PAH), so we were underpowered to assess whether a transcriptional signature is associated with disease outcomes, although this is a focus of future studies. Lastly, we acknowledge that our observations need to be confirmed and developed beyond the transcript level. While we have started to pair sequencing results with functional assessments of potential importance (e.g., replication competency and anoikis-resistance) additional work needs to explore mechanisms by which transcription changes inform vascular cell dysfunction and can be rescued with treatment.

In conclusion, we have demonstrated the validity and promise of using PAECs cultured from routine RHC balloon tips in living patients with PAH. To our knowledge, this “cell biopsy” approach is a first-in-field window into dynamic signatures during the PAH disease course that may be harnessed to refine therapeutic selection. Our findings demonstrate that these cells provide a consistent and reproducible transcriptome profile that distinguishes between patients with and without pulmonary vascular disease, heralds disease progression (and remission) and recapitulates established and emerging pathways of interest. We provide evidence of anoikis-resistance as a novel paradigm for endothelial proliferation in PAH. This method provides an available source of cells that can be repeatedly characterized for deep phenotyping over the disease course, a long-standing barrier to translational research in pulmonary vascular disease.

## Methods

### Study sample

All patients referred to the Rhode Island Hospital Pulmonary Hypertension Center undergoing RHC for the purposes of pulmonary vascular disease evaluation and management were eligible. The RHC procedure was not altered for the purposes of the study. The study was approved by the Lifespan Institutional Review Board (IRB #016311 and #001218), informed consent was obtained from all participants and all methods were performed in accordance with the relevant guidelines and regulations. The subjects included here with biological replicates were re-approached and notified of our intent to feature their clinical trajectory (which may be identifiable by history) and serial sequencing and signed an additional consent for publication.

All patients were initially evaluated by a PH clinician, and the need for RHC was based on clinical indications. Repeat RHCs (from which biological replicates were obtained) were performed at the discretion of the treating clinician. The initial clinical diagnosis of PH was made by the treating clinician. Clinical phenotyping was confirmed retrospectively by author N.S. who was blinded to sequencing data. A clinical phenotype was assigned after all available clinical and hemodynamic data was incorporated and based on the World Symposium on PH clinical classification^11^. When hemodynamic values at the time of RHC with PAEC sampling conflicted with the clinical PH diagnosis (e.g., pulmonary capillary wedge pressure [PCWP] above 15 mmHg without evidence of left heart disease, pulmonary vascular resistance [PVR] below 3 Wood units in treated PAH patients), the prior clinical diagnosis and clinical data were prioritized for Group designation, and index RHC at diagnosis (but not necessarily PAEC sampling) was confirmed to agree with standard hemodynamic definitions^11^. Precapillary PH in patients with a non-Group 1 clinical diagnosis (e.g., left heart or lung disease) was defined as a mean pulmonary artery pressure (mPAP) > 20 mmHg, a PCWP ≤ 15 mmHg, and a PVR ≥ 3 Wood units. Those subjects with a clinical indication for a RHC but who were determined not to have PH based on a resting mPAP < 20 mmHg and did not meet criteria for exercise-induced PH (total pulmonary resistance < 3 mmHg per liter of cardiac output with exercise)^57^ were designated as controls. Patients with exercise-induced PH were not included in our analysis of PAH patients based on prior classification guidelines^58^ and given this is an area of controversy. Medical records or our research registry were reviewed for clinical data, which was collected at the time of or as close as possible to (within 6 months) of RHC.

### Retention of pulmonary artery catheter (PAC) balloon tips and primary culture

Detailed methods have been previously published and we have not altered the protocol since the original publication^4^. Briefly, at the end of the RHC procedures, PACs were retracted into the catheter sheath and both catheter and sheath were removed from the patient. The tips of the catheters were then advanced out of the sheath and placed directly into warm media (37 °C) (EndoGRO; Millipore Sigma, Billerica, MA) with the balloon deflated and immediately into a heater for travel to the laboratory. The PAC tips with balloon were placed directly into one well of a 24 well plate with Attachment Factor Solution (Cell Applications, Inc; San Diego, CA) and washed with fresh media (EndoGRO-vascular endothelial growth factor [VEGF] complete media kit; Millipore Sigma, Billerica, MA) every 2 days. Cells were then seeded into T-25 and T-75 flasks until passaged cells reached confluence over the next 4–5 days. We have confirmed EC phenotype through passage eight with these methods, as previously described^4^. PAECs were directly characterized or frozen at passage 3–4. For this study, all RNA sequencing was performed on passage 3–4 for all samples; passage number was fixed for analysis of biological replicates and clinical subgroups. Only the PH clinician conducting the RHC was aware of patient characteristics. The remainder of the study staff were blinded.

### RNA sequencing and analysis

Library preparation and RNA sequencing was conducted through Genewiz (Cambridge, MA). All successfully cultured PAECs harvested between December 2016 and January 2021 were included. Specimens were submitted for sequencing in two batches and grouped randomly within each batch. Libraries were sequenced using a 2 × 50 bp paired end rapid run on the Illumina HiSeq2500 platform. Each batch was sequenced all at one time and equally distributed across sequencing lanes. Technical replicates were not included. Quality control on sequencing data was performed using FastQC (Babraham Bioinformatics, Cambridge, UK). Raw reads were analyzed using unsupervised dimensionality reduction via Principal Component Analysis (PCA) and clustering to characterize fold changes across serial passages (passage 3 versus 4), across biological replicates and clinical subgroups (fixed passage numbers; Group 1 PAH vs. controls and Group 1 PAH vs. precapillary PH in Groups 2–5 PH). P-values were adjusted for multiple comparisons using a Bonferroni-type method based on a false discovery rate < 0.05^59^. A p_FDR_ < 0.05 was considered significant. Reactome and Hallmark pathway analysis of the top differentially expressed genes in PAH as compared to controls was performed using DAVID bioinformatics tools^60^.

In patients with biological replicates, the clinical course informed our approach. Instead of an unsupervised analysis, we focused on investigating potential mechanisms of disease by a priori selecting gene lists known to correlate with disease processes. Genes relating to PoPH were selected based on established lists in the literature^14^ and for PVOD (including *EIF2AK4*, the causative gene^49^) and the gene ontology (GO) Pathway^61^ for hypoxia (GO: 0071456), a characteristic hallmark in PVOD. The DAVID database was used to cluster differentially expressed genes by GO biologic processes, including genes related to anoikis (GO: 0043276).

### Single cell and population growth, adhesive forces and characterization of anoikis

Once cells reached confluence, fresh (never frozen) PAECs were shipped in media at ambient temperature to the Stevens’ laboratory and cells were expanded. Commercial controls were obtained from Lonza (Basel, Switzerland).

#### Single-cell cloning

Single-cell clonogenic assays were performed as described elsewhere^62, 63^. Cells were trypsinized, transferred to flow cytometry tubes containing standard culture media (EndoGRO-VEGF complete media kit; Millipore Sigma, Billerica, MA) at 5 × 10^5^ cells/tube. Cells were typically seeded at single-cell density on four 96-well plates containing 200 μL/well of complete media (EndoGRO-VEGF complete media kit; Millipore Sigma, Billerica, MA) and 1% penicillin–streptomycin using a BD FACS Aria II flow cytometer. Cells were incubated at 37 °C with 5% CO_2_-room air for 14 days without a media change. Media was checked on the wells and if needed, media was added on the 14th day. On the 14th and 28th days, each well was examined by light microscopy to assess colony size and representative wells were photographed.

#### Dilutional cloning

For dilutional cloning, PAECs were trypsinized and passed through a cell strainer and were counted using the Countess Automated Cell Counter (ThermoFisher Scientific). Then, cells were seeded on 24-well plates with densities ranging from 125,000 to 62 cells/well (1:2 dilutions) in 12 different wells containing 1 mL/well of complete media (EndoGRO-VEGF complete media kit; Millipore Sigma, Billerica, MA) and 1% penicillin–streptomycin. Cells were incubated at 37 °C with 5% CO_2_-room air for 10 days without a media change. On the 5th and 10th day, each well was examined by light microscopy to assess colony size and representative wells were photographed for the quantification of confluency.

#### Anoikis assay

To determine anoikis resistance, PAECs were trypsinized, counted, and seeded on ultra-low attachment 6-well plates (Corning) with a density of 1 × 10^6^ cells/well. Cells were incubated at 37 °C and 5% CO_2_ for 1, 3 and 7 days. Viability and cell counts were assessed after 1, 3, and 7 days by Propidium Iodide and Annexin V staining (ThermoFisher Scientific, Cat # V13242) with the use of flow cytometry. Proliferative capacity was tested by re-seeding cells on regular attachment culture dishes. Cell growth was tracked every other day until confluence was reached. Each cell type was evaluated three times, at passages ranging from 3 to 8.

#### Lectin affinity

PAECs were grown to confluence on gelatin-coated 6-well plates. The cells were trypsinized and triturated to assure single-cell suspensions, then resuspended in PBS. Cells were centrifuged (212×*g*) for 5 min and the cell pellets were resuspended in serum-free media (EndoGRO without supplement). 2.5 × 10^5^ cells were kept in the bottom of the glass 6-well plate, along with 2 mL of serum-starved media. *Helix pomatia* and *Griffonia simplicifolia* lectins were diluted 1:1000 and added to each well, and then cells were examined by light microscopy to assess for agglutination. Representative wells were photographed.

#### Image analysis

All images were taken using a Nikon Eclipse T*s*2 light microscope with a 10 ×/0.25 Ph1 DL objective. For the clarity of the images two different filters were used, including an Enhance Local Contrast filter and a bandpass filter from Fiji ImageJ.

#### Statistical analysis

Numerical data are reported as mean ± SD. One-way ANOVA was used to evaluate differences between cells within patient groups, with a Friedman multiple comparison post hoc test, as appropriate. Two-way ANOVA was used to evaluate differences between cells obtained from different patient groups and day variability, with Tukey’s multiple comparison post hoc test. Significance was considered p < 0.05.

### Supplementary Information

Supplementary Information.

## Data Availability

The datasets generated and analyzed during the current study were deposited to the GEO repository (GSE243193).
