# Supplementary material for: Transcriptional profiles of pulmonary artery endothelial cells in pulmonary hypertension
Source: Sci Rep. 2023 Dec 18;13:22534. doi: 10.1038/s41598-023-48077-6 (PMC10728171; doi:10.1038/s41598-023-48077-6)
Supplement: Supplementary file 1 — Supplementary Information. [file 41598_2023_48077_MOESM1_ESM.docx]

**Transcriptional Profiles of Pulmonary Artery Endothelial Cells in Pulmonary Hypertension**

Navneet Singh, Carsten Eickhoff, Augusto Garcia-Agundez, Paul Bertone, Sunita S. Paudel, Dhananjay T. Tambe, Leslie A. Litzky, Katherine Cox-Flaherty, James R. Klinger, Sean F. Monaghan, Christopher J. Mullin, Mandy Pereira, Thomas Walsh, Mary Whittenhal, Troy Stevens, Elizabeth O. Harrington, and Corey E. Ventetuolo

| **Table S1. Subject-level characteristics by clinical subgroup*** | | | | | | | | |
| --- | --- | --- | --- | --- | --- | --- | --- | --- |
|  | | | **Group 1 PAH^†^** | **Group 2 PH** | **Group 3 PH** | **Group 4 PH** | **Group 5 PH** | **Control** |
| No.^‡^ | | | 20 | 18 | 8 | 1 | 4 | 3 |
| Age, yr | | | 57 (30 – 83) | 74 (40 – 91) | 69 (46 – 82) | 56 | 42 (33 – 71) | 53 (19 – 78) |
| Female sex | | | 17 (85) | 12 (63) | 2 (25) | 0 | 0 | 3 (100) |
| BMI, kg/m^2^ | | | 30 (16 – 45) | 28 (23 – 46) | 30 (16 – 37) | 28 | 30 (26 – 40) | 22 (21 – 31) |
| Hemodynamics | | |  | | | | | |
|  | | RAP, mmHg | 7 (3 – 14) | 14 (7 – 24) | 7 (2 – 11) | 5 | 8 (5 – 24) | 7 (1 – 7) |
|  | | mPAP, mmHg | 37 (16 – 62) | 38 (23 – 57) | 35 (27 – 43) | 32 | 41 (31 – 50) | 18 (16 – 21) |
|  | | PCWP, mmHg | 11 (5 – 17) | 20 (9 – 28) | 12 (7 – 18) | 9 | 12 (6 – 15) | 10 (8 – 12) |
|  | | CO, L/min | 5.7 (2.4 – 12.9) | 4.7 (2.6 – 10.8) | 4.6 (2.7 – 6.9) | 4.8 | 8.8 (4.5 – 11.6) | 5.3 (4.9 – 5.6) |
|  | | PVR, wood units | 4.0 (1.2 – 19.6) | 2.7 (1.1 – 8.8) | 4.6 (2.3 – 9.2) | 4.8 | 2.7 (2.2 – 9.8) | 1.8 (1.1 – 1.9) |
| 6-minute walk distance | | | 390 (150 – 600) | − | − | − | − | − |
| Functional Class | | |  | | | | | |
|  | | *no. available* | 17 | − | − | − | − | − |
|  | | I or II | 15 (75) | − | − | − | − | − |
|  | | II or III | 2 (10) | − | − | − | − | − |
| BNP, pmol/dL | | | 46.5 (15 – 1342) | − | − | − | − | − |
| PAH treatment naïve | | | 6 (30) | − | − | − | − | − |
| Combination treatment | | | 9 (45) | − | − | − | − | − |
|  | PDE5 | | 11 (55) | 2 (11) | − | − | 1 (25) | − |
|  | ERA | | 7 (35) | − | 1 (13) | − | 1 (25) | − |
|  | Prostacyclin | | 9 (45) | − | − | − | − | − |
| Data represented as n (%) or median (range). *Subgroup as determined by NS based on clinical criteria and pulmonary hypertension consensus definitions^1^, blinded to sequencing results. Hemodynamic values at the time of right heart catheterization (RHC) may have been outside of range for Group due to the inclusion of prevalent patients and temporal changes with treatment. In these cases, the clinical diagnosis at presentation and RHC at diagnosis was prioritized for Group designation. ^†^Excludes exercise-induced PH. **^‡^**Unique catheter tips. PAH=Pulmonary Arterial Hypertension. PH=Pulmonary Hypertension. BMI=body mass index. RAP=right atrial pressure. mPAP=mean pulmonary artery pressure. PCWP=pulmonary capillary wedge pressure. CO=cardiac output. PVR=pulmonary vascular resistance. BNP=brain natriuretic peptide. PDE5=phosphodiesterase type 5 inhibitor. ERA=endothelin receptor antagonist. | | | | | | | | |

| **Table S2. Similarity of Passaged Specimens** | | | | |
| --- | --- | --- | --- | --- |
| **Pair** | **PH Diagnosis** | **Pair similarity** | **Average similarity, all others** | **Similarity, closest other** |
| **EC003, passage 3**  **EC003, passage 4** | Portopulmonary hypertension | 0.991 | 0.849 | 0.973 |
| **EC031, passage 3**  **EC031, passage 4** | Group 2 pulmonary hypertension | 0.954 | 0.794 | 0.953 |
| **EC063, passage 3**  **EC063, passage 4** | Heritable PAH | 0.978 | 0.856 | 0.973 |
| PH=pulmonary hypertension; PAH=pulmonary arterial hypertension. | | | | |

| **Table S3. Clinical Characteristics of Single Cell Cloning Specimens** | | | | | | | |
| --- | --- | --- | --- | --- | --- | --- | --- |
| **Specimen** | **PH Diagnosis** | **PAH Medications** | **RAP, mmHg** | **mPAP, mmHg** | **PCWP, mmHg** | **CO, L/min** | **PVR, Wood units** |
| EC063 | HPAH | Treprostinil 3.25mg TID, Tadalafil 40mg daily, Macitentan 10mg daily | 7 | 49 | 10 | 5.0 | 7.8 |
| EC074 | PVOD | N/A | 15 | 47 | 11 | 1.9 | 18.9 |
| EC079 | IPAH (1) | N/A | 19 | 69 | 13 | 3.3 | 16.8 |
| EC085 | IPAH (2) | Tadalafil 40mg daily | 11 | 31 | 14 | 5.2 | 3.3 |
| PH=pulmonary hypertension. HPAH=heritable pulmonary arterial hypertension. PVOD=pulmonary veno-occlusive disease. IPAH=idiopathic pulmonary arterial hypertension. TID=three times daily. N/A=not applicable | | | | | | | |

**Figure S1.** Heatmaps of passaged specimens from subjects with **A.** Portopulmonary hypertension **B.** Group 2 pulmonary hypertension and **C.** heritable pulmonary arterial hypertension demonstrating no significant transcriptomic shift over serial passaging.


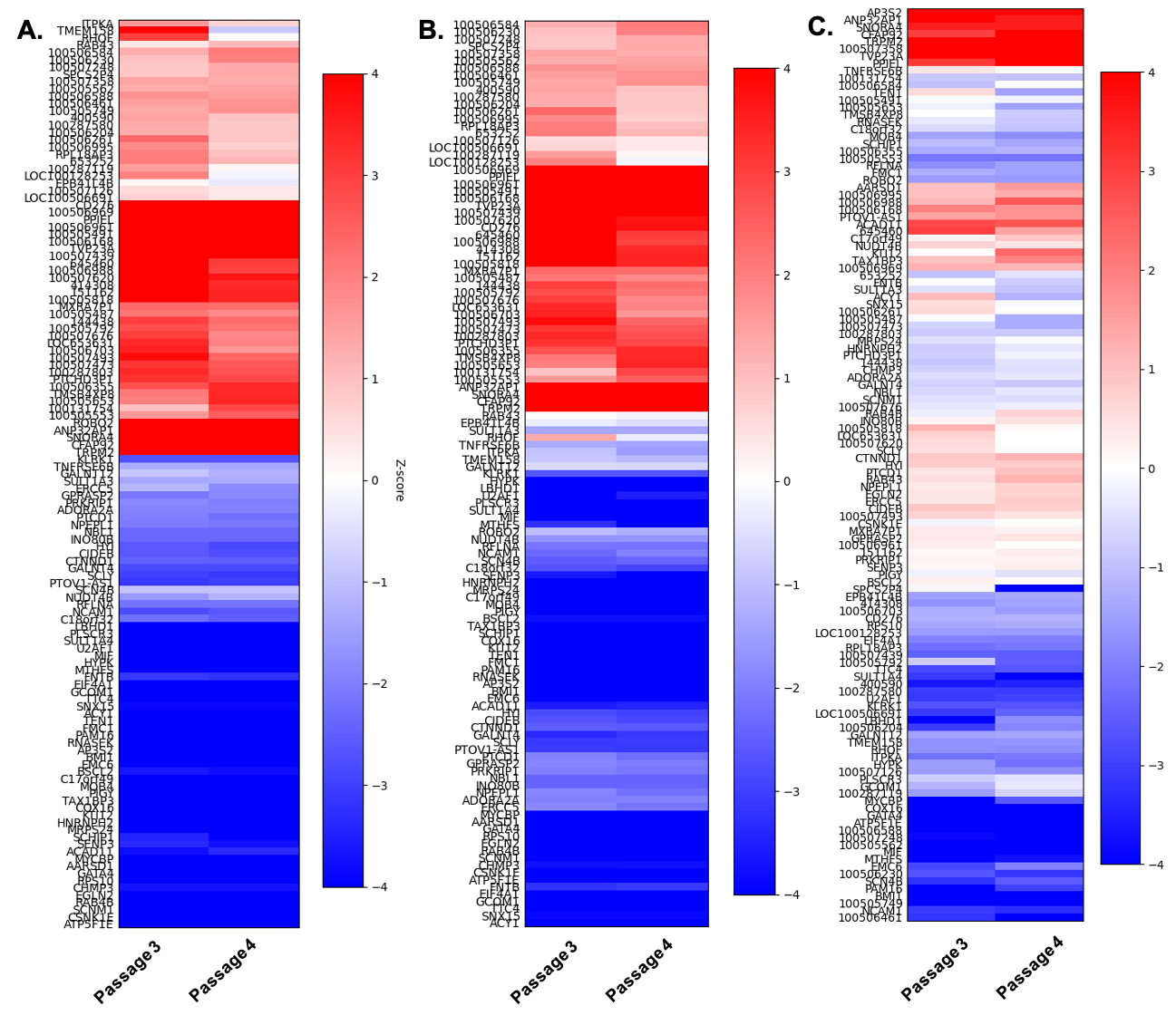


**Figure S2.** Differential gene expression in a second patient with hereditary pulmonary arterial hypertension (HPAH2) with an *ALK1* mutation and hereditary hemorrhagic telangiectasia. **(A)** Volcano plot showing that a patient with *AKL1* HPAH (HPAH2) had no significant differential gene expression when compared to controls (unsupervised analysis). **(B)** Heatmap of differential gene expression demonstrates largely equivocal gene expression in this subject compared to controls.


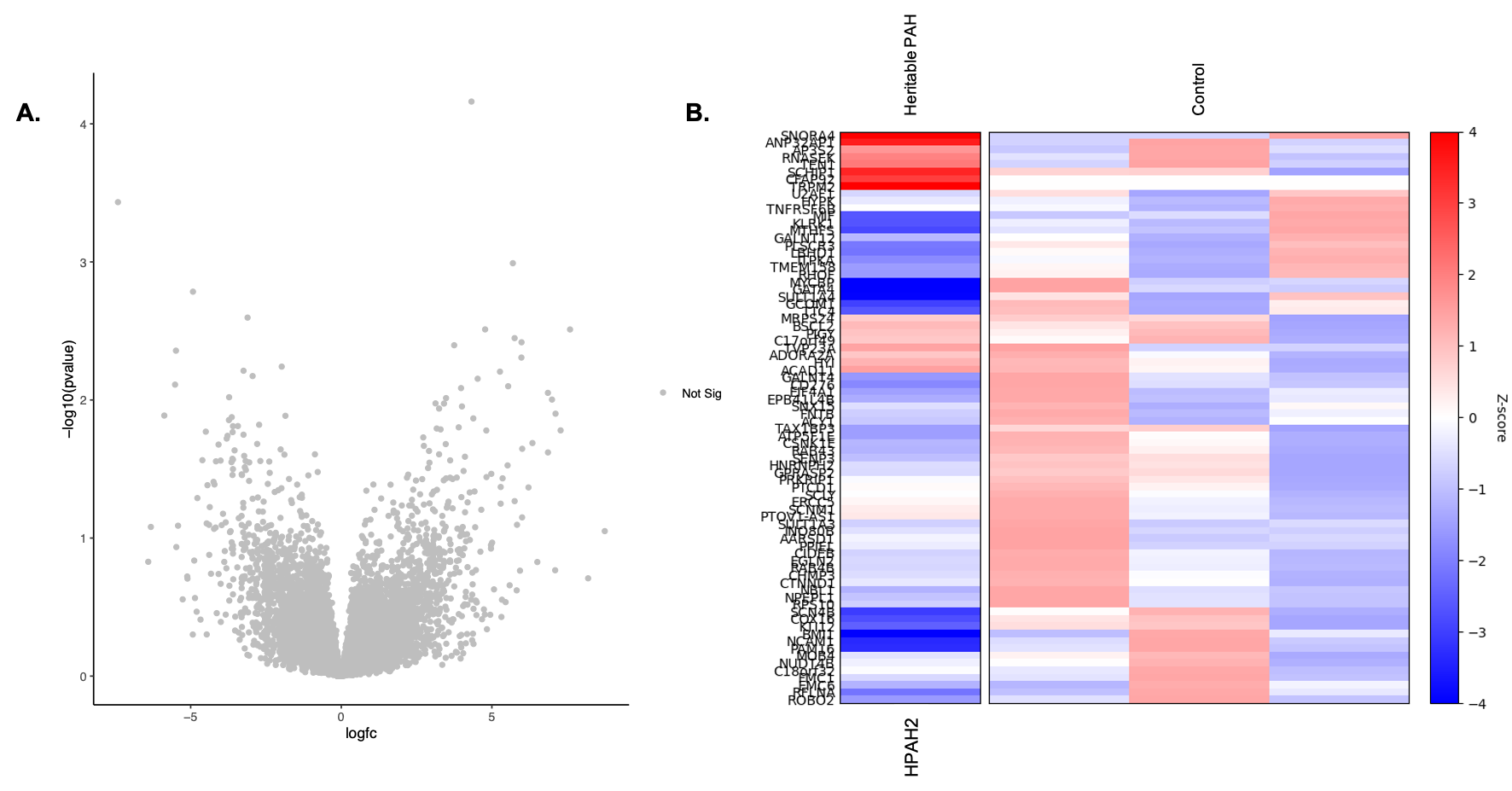


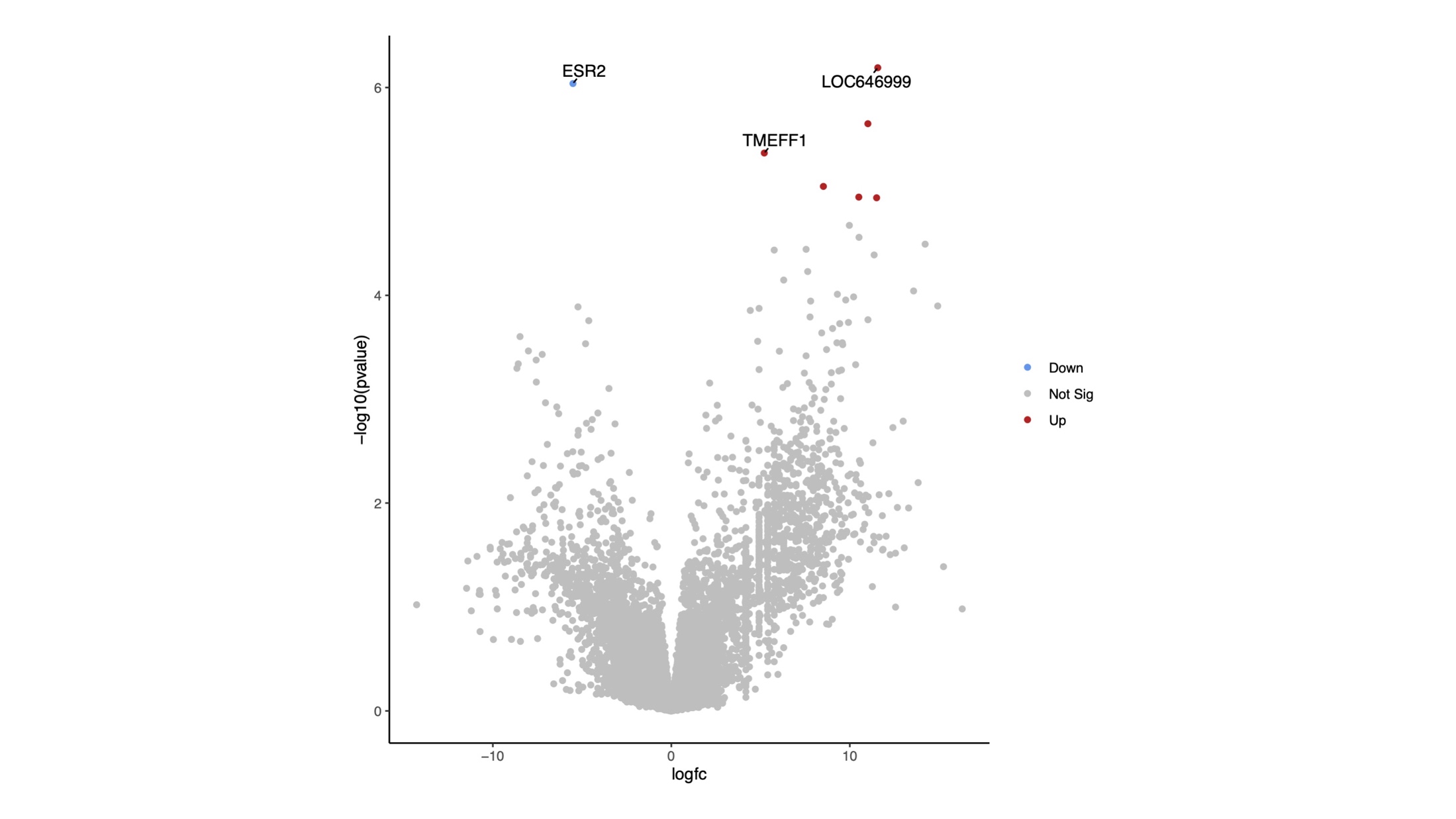


**Figure S3.** Volcano plot of unsupervised fold-change analysis comparing two patients with hereditary pulmonary arterial hypertension (HPAH) demonstrates significantly different gene expression suggesting heterogeneity between these two HPAH subjects.

1. Simonneau G, Montani D, Celermajer DS, Denton CP, Gatzoulis MA, Krowka M, Williams PG, Souza R. Haemodynamic definitions and updated clinical classification of pulmonary hypertension. *European Respiratory Journal*. 2019;53.
